# Experiences of patients advising on mental health research: qualitative study in South-East European countries

**DOI:** 10.1192/bjo.2021.1077

**Published:** 2022-01-10

**Authors:** Selman Repišti, Tihana Majstorović, Fjolla Ramadani, Mirjana Zebić, Ljubisha Novotni, Nikolina Jovanović

**Affiliations:** Psychiatric Clinic, Clinical Center of Montenegro, Montenegro; Association for Mental Health ‘Menssana’, Bosnia and Herzegovina; School of Psychology, University of Prishtina, Kosovo; Clinic of Psychiatry, University Clinical Centre of Serbia, Serbia; University Clinic of Psychiatry-Skopje, North Macedonia; Unit for Social and Community Psychiatry, WHO Collaborating Centre for Mental Health Services Development, Queen Mary University of London, UK

**Keywords:** Mental health, patient involvement, qualitative research, patient participation, Eastern Europe

## Abstract

**Background:**

Although patient and carer involvement in research is well-developed in many countries, this area has been largely overlooked in South-East European countries.

**Aims:**

To explore experiences of patients participating in newly set up lived experience advisory panels (LEAPs) within a European Commission funded, large-scale, multi-country mental health research project that focused on improving treatment of individuals with psychosis.

**Method:**

Twenty-one mental health patients were individually interviewed across five countries: Bosnia and Herzegovina, Kosovo, Montenegro, North Macedonia and Serbia. Topic guides covered the experience of participating in LEAPs and their sustainability. Data were analysed by framework analysis.

**Results:**

Seven themes were identified about participating in LEAPs: predominantly positive evaluation, high levels of participant motivation, therapeutic benefits for participants, few challenges, various future perspectives, positive appraisal of the research project and mixed reflections on mental healthcare. Overall, patients’ experiences were positive and enabled them to feel empowered. Patients expressed interest in remaining involved in advisory panels. Additionally, they felt that they could potentially contribute to the work of non-government organisations.

**Conclusions:**

This study is among the few studies exploring patient participation in research projects, and the first such study conducted in South-East European countries. Patients are highly motivated for this engagement, which has the potential to empower them to take on new social roles. Significant efforts at the national level are needed in each country, to make patient involvement in research standard practice.

Patient involvement in research has the potential to improve the quality, relevance and impact of health research, as well as the transparency of the process and the accountability of the wider community of researchers.^1,2^ Additionally, involvement of patients of mental health services and caregivers in research projects can improve quality of research activities, filter out irrelevant research questions, empower patients to actively contribute to improvement of services, reduce stigma and support meaningful dissemination.^3,4^ Recruitment strategies have also been shown to be more successful if designed in collaboration with patients.^5^ Patients who are involved in research also help to ensure that the interventions are patient-friendly.^6^ Nevertheless, patient involvement in South-East European (SEE) countries is in early stages of development, and patient involvement in research is practically non-existent. Indeed, patients in this region may not be even familiar with the concept of patient involvement. The present study was conducted as part of a large, 3.5-year research project Implementation of an effective and cost-effective intervention for patients with psychotic disorders in low and middle-income countries in South Eastern Europe (IMPULSE), funded by the European Commission (grant number 779334), to improve treatment and care of people with psychosis in five SEE countries: Bosnia and Herzegovina, Kosovo, Montenegro, North Macedonia and Serbia.^7^ The IMPULSE study focused on involving mental healthcare of patients and caregivers in all stages of the study, and to our knowledge, this was the first time that mental health patients were invited to advise on research in this region. The current study was created to explore patients’ experiences of participating in newly set up lived experience advisory panels (LEAPs) within the IMPULSE study.

## Method

### Research design

Semi-structured, in-depth individual interviews were conducted to enable exploration of participants’ views and experiences. Data were analysed with framework analysis.^8^ The Consolidated Criteria for Reporting Qualitative Research (COREQ) were used to report key aspects of the qualitative study.

### Overview of LEAP meetings

The LEAP meetings were organised every 3 months for 18 months, in a neutral space outside of hospitals and mental healthcare institutions; except for Montenegro, where the first meeting was organised within the Clinical Center of Montenegro but outside its Psychiatric Clinic, and North Macedonia where the first two meetings were held in the University Psychiatric Clinic in Skopje. In total, five meetings were organised per each country. Each LEAP included six to eight members who were patients recruited from services participating in the IMPULSE study. Gender, age and experience of using services were taken into account when selecting the LEAP members, to ensure diversity of perspectives. Two weeks before each meeting, LEAP members were provided with written materials about the topics that would be discussed during the meeting. On average, the meetings lasted 80 min.

### Sample

All 28 LEAP members were invited to participate in interviews, and 21 accepted. Four people in Kosovo and three in Macedonia refused because they were not interested (*n* = 1), did not want to speak over the phone (*n* = 1), did not want to be recorded (*n* = 2), moved to another country (*n* = 1) or did not answer (*n* = 1). In three countries (Bosnia and Herzegovina, Montenegro and Serbia) all LEAP members (five in each country) were interviewed.

### Topic guides

The topic guides included the following: introduction; experience of participating in LEAP as part of a research project (personal like/dislike of conversations, LEAP's contribution to the project, benefits, what needs to change/improve, challenges, meeting materials); sustainability and future aspirations (continuous involvement, other activities, group development) and other comments. Topic guides are shown in Supplementary Appendix 1 available at https://doi.org/10.1192/bjo.2021.1077. The guides were created first in English, in collaboration with experts in qualitative research. Next, they were translated into local languages of the participating countries. Finally, the translations were discussed with the whole team and the final versions were created. Researchers were trained to use specific prompts to explore a topic more deeply or to further obtain information when needed.

### Procedure and data collection

Researchers in each country contacted their LEAP members and those who agreed to be interviewed were invited to sign the consent forms. However, because most of the countries were in a total or partial lockdown because of COVID-19, not all patients could sign the consent forms. Instead, they gave verbal approval (which was witnessed and recorded) and signed the consent forms later. The authors assert that all procedures contributing to this work comply with the ethical standards of the relevant national and institutional committees on human experimentation and with the Helsinki Declaration of 1975, as revised in 2008. All procedures involving human participants were approved by the Ethics Committee of the Clinical Center of the University of Sarajevo, Bosnia and Herzegovina (approval number 03-02-47500, approved on 13 September 2018); Komisioni Etiko Profesional, Hospital and University Clinical Service of Kosovo, University Clinical Center of Kosovo, Kosovo (approval number 904, approved on 8 June 2018); Ethical Committee for Research with Humans, Medical Faculty at the University of Cyril and Methodius in Skopje, North Macedonia (approval number 03-2237/12, approved on 21 May 2018); Ethics Committee of the Clinical Center of Montenegro, Montenegro (approval number 03/01-11066/1, approved on 19 July 2018) and Ethical Committee of the University of Belgrade Faculty of Medicine (approval number 2650/V1-3, approved on 26 June 2018). Research team and reflexivity is thoroughly described in Supplementary Appendix 2.

After collecting consent from participants, the interview was scheduled for a convenient time for each participant. At the beginning of the interview, participants were reminded about the aim of the interviews, that the participation is voluntary and that they were free to stop the interview at any time without any consequence. They were also assured about confidentiality and informed that the session would be audio-recorded. In addition, participants were informed of what would happen with the data gathered from the interview and how interviews work. Participants were compensated with €25 for their participation. The majority of semi-structured, in-depth individual interviews were conducted through telephone calls (*n* = 17), and four interviews were conducted in-person at the patient-led organisation Menssana in Sarajevo, Bosnia and Herzegovina. All interviews were done in local languages, audio-recorded and transcribed verbatim by professional services. The audio recordings were deleted after verbatim transcription, and no field notes (e.g. on non-verbal cues) were taken during or after the interviews. The transcripts were not returned to participants for correction for two reasons: most participants did not have email addresses and we could not deliver the hard copies of the transcripts because of COVID-19 lockdown conditions.

### Analysis

Data were analysed primarily with an inductive approach to framework analysis,^8^ which presented a purely qualitative account of data, whereas the content analysis included counting of some words, codes, categories, etc. First, one transcript for each of the five countries was translated into English. Researchers familiarised themselves with the translated transcripts and each researcher created a preliminary coding list. The individual preliminary coding lists were then combined in a single coding list. Second, researchers coded all of the remaining untranslated transcripts in their respective languages to check the coding list and identify additional codes; additional codes were translated into English for the final coding list. The final coding list was created by consensus through group discussions.

Next, the codes were grouped to develop broad framework categories based on concerns related to the research question and other concerns emerging from the familiarisation stage (see Supplementary Appendix 3). The data were then organised according to the framework categories and summarised. This was followed by mapping and interpretation of the data to find patterns. This process was ongoing until no additional patterns were found. The LEAP members in each country provided feedback on the preliminary findings.

## Results

The study included 21 individuals across five countries (Table 1). Participants’ mean age was 49.2 years and there were more men (*n* = 12) than women (*n* = 9). Fifteen participants were single and six were married or in a relationship. Eleven participants completed secondary education and the remaining ten had obtained a college and/or university degree. More than half of the participants were unemployed (*n* = 12). On average, the shortest interviews were held in Kosovo (median 13.3 min) and the longest ones were held in North Macedonia (median 24 min). In total, 21 interviews were conducted and the same number of transcripts was analysed for the study. The list of codes and categories is shown in Supplementary Appendix 3. Seven themes were identified (summarised in Supplementary Table 1).

**Table 1:** Study participant demographics

	All	Bosnia and Herzegovina	Kosovo	North Macedonia	Montenegro	Serbia
	*N* = 21	*n* = 5	*n* = 3	*n* = 3	*n* = 5	*n* = 5
Age, mean	49.2	58.0	40.6	44.0	50.0	47.8
Gender						
Male	12	1	3	2	3	3
Female	9	4	0	1	2	2
Marital status						
Single	15	5	2	1	3	4
In a relationship/married	6	0	1	2	2	1
Education						
Primary and secondary school	11	2	3	2	4	0
College and university	10	3	0	1	1	5
Employment						
Employed	9	1	3	2	2	1
Unemployed	12	4	0	1	3	4

### Theme 1: participants’ views on the LEAP meetings

This was the first ever experience of participating in LEAPs for all participants, and their general impressions were largely positive:

‘Well, I'm satisfied. There are a couple of nice people, uh, you as researchers are very dedicated and it is perfectly clear to us everything you ask from us.’ (Female, 43 years old, Montenegro)

‘Everything was good. Some things were on point.’ (Male, 37 years old, Kosovo)

The participants particularly emphasised that the received materials were clear and easy to comprehend:

‘Quite clear enough. You also clarify them. So, you read every material to us, so explain to us everything that is not clear, so that we are literally instructed in those materials perfectly.’ (Female, 50 years old, Montenegro)

‘I think that the materials are clear and concise. They are short and easily understandable. The main points are well explained and described.’ (Female, 39 years old, North Macedonia)

When discussing the frequency of meetings, many suggested the meetings should be held more frequently (e.g. every month), although some people were in favour of meeting when needed or if there are specific materials to be discussed:

‘Well I mean, maybe those meetings should be more frequent, I don't know, to be more times a year than there are like this.’ (Male, 52 years old, Serbia)

‘[The meeting should be held] … Well, maybe monthly.’ (Male, 60 years old, Montenegro)

‘At some of the previous meetings most of the people said there was a need for more frequent meetings. If you and your team would prepare yourselves and have more material that could be presented, our feedback would be useful, otherwise I don't see the point in having more meetings if we don't have what to discuss at those meetings.’ (Male, 33 years old, Macedonia)

### Theme 2: drivers for LEAP involvement

Although most LEAP members said they were highly motivated to attend and participate in LEAP meetings, some people had some issues with motivation. LEAP members were motivated by extrinsic factors (be a part of international study, be in contact with kind and respectful mental health professionals (LEAP leaders), receive money and exchange useful and practical experiences with group of other patients) and intrinsic factors (be asked for their opinion, help others, use their experience for something good, therapeutic effects of the group and have something new and inspiring in life):

‘ …. Yes, and it was also about the money.’ (Male, 50 years old, Montenegro)

‘At every meeting all the members have enough time to speak up, to give opinions and suggestions … I feel I can express myself.’ (Male, 33 years old, Macedonia)

Identified barriers included physical factors (psychomotor restlessness owing to illness or therapy; lack of concentration, especially if people talk at the same time; fear of taking on long-term obligations because of their illness) and challenges of the new role (lack of confidence in their (further) contribution to the study, lack of further inspiration):

‘I personally find it difficult to be able to [continue], since I am still in a regular employment relationship and I could not come to recite some short meetings.’ (Female, 54 years old, Bosnia and Herzegovina)

### Theme 3: therapeutic benefits of participating in LEAPs

The participants found the meetings very beneficial, largely in relation to their own recovery. The participants benefited from regularly meeting and socialising with peers, they were keen to discuss their mental health problems and treatment options with others, and they felt less anxious and happier after the meetings. The bonding between the members grew over time, and some of them were socialising in between meetings. The researchers felt that the participants often saw meetings as therapeutic sessions rather than advisory panels:

‘Compared to how I was before, I feel better now; physically and mentally.’ (Male, 37 years old, Kosovo)

‘Well, I felt comfortable in that company and then I accepted that [LEAP meetings] … as some kind of … some kind of commitment to help me heal. And I felt kind of nice in that group, it was nice, simple and those who came from Koševo Clinic [LEAP leaders], they kind of empowered me, I just felt nice.’ (Female, 61 years old, Bosnia and Herzegovina)

‘Well, I think … that fills with positive energy, that you are all positive, from the psychiatrist to you, all the co-workers … you just understand us, you know our problem … You support us.’ (Female, 50 years old, Montenegro)

The participants also appreciated being part of an international study, having the opportunity to voice their concerns, feeling useful, being treated respectfully by researchers and earning money for their effort and experience. This new social role had an empowering effect on some of the interviewed participants, and stands out as something that is relevant in the context of deinstitutionalisation, community psychiatry and resocialisation of people with psychotic disorders.

‘[It is very important to me] to be asked for an opinion. Because, as a patient, you are always in a position like – “Here is your therapy”, and no one has enough time to deal with you. And the fact is that these patients need some attention.’ (Female, 61 years old, Bosnia and Herzegovina)

‘Well, the benefit is on the one hand that it can contribute to the improvement of mental health through these groups the benefit is that the one who participates in it in a way gains a little more self-confidence because it can be useful in some way, in a way at least a little can within its some life possibilities, abilities, etc. I liked that, for example, that there is an equal relationship between the doctor and the patients.’ (Female, 45 years old, Serbia)

‘Well if there was no possibility in participating, I wouldn't have known what it's about. Maybe it would have been conducted in a private health facility and then we could immediately give an opinion and it would be taken into consideration that we are an important part in creating the politics, because there are no doctors without patients and there are no patients without doctors.’ (Male, 33 years old, North Macedonia)

The LEAP members highlighted that in meetings they were given the opportunity to talk freely about their mental health issues and other topics:

‘At every meeting all the members have enough time to speak up, to give opinions and suggestions because that is what we should do. I feel I can express myself.’ (Male, 33 years old, Macedonia)

### Theme 4: challenges to participating in LEAPs

The majority of participants did not identify challenges related to their membership in the LEAP. However, among identified perceived challenges, the most prominent were issues with personal mental health (e.g. emotional responsiveness, passivity, scepticism, anxiety) or physical health (e.g. disability):

‘There were no challenges.’ (Male, 40 years old, Kosovo)

‘I thought it was something much more complicated than it is … that it will go public, that we will be, uh, identified, It was a little bit of scepticism. But then when I saw it, it was done at discretion.’ (Female, 50 years old, Montenegro)

### Theme 5: various future perspectives of LEAP meetings

The participants were largely interested in remaining involved in this or similar panels/groups. The members felt that they could potentially contribute to the work of a non-government organisation. It was also suggested that members could attend online meetings (e.g. via Zoom, Skype, etc.).

‘My recommendation is to keep on working after the Coronavirus is over. I plan to form an association with several people and to continue working because it is obvious that that provides good results.’ (Male, 60 years old, North Macedonia)

‘Yes [I would like to see the LEAP group growing]! To communicate with each other, to form a bigger group. To have fun.’ (Male, 40 years old, Kosovo)

### Theme 6: positive appraisal of the research project

The participants were largely positive about the research project and the digital mental health intervention (DIALOG+) studied in the IMPULSE study. Positive aspects included terms and phrases such as ‘effective’, ‘interesting’, ‘useful’, ‘relaxing’ and ‘sustainable’. The intervention was seen as preferable compared with existing routine psychiatric appointments:

‘The project is quite interesting. I really like that it is more guided towards dialogue that is well thought out. The questionnaires provide relevant answers and research. I like the entire concept. We fit in well in the group and everyone gives ideas, shares their opinion, and I believe that everyone has contributed to the improvement of the entire project, and in the end, we got a final product that is functional and serves the patients.’ (Female, 39 years old, North Macedonia)

‘I liked the project because it has to do with my life [as a patient].’ (Male, 37 years old, Kosovo)

‘Well, I really like the DIALOG+ project, because of the questions that are asked to the patient himself, so his mental and physical condition, his feelings, everything, everything, everything, which is very important in the very psychiatric moment of the condition, is taken into account.’ (Female, 53 years old, Serbia)

### Theme 7: mixed reflections on mental healthcare

The participants shared their views of mental healthcare services and professionals, and expressed a variety of positive, neutral/mixed or negative views:

‘Literally, the visits taking place and sleeping, lying in the room and taking therapy and sitting there in the hallway, talking and something. When I was here last time, in the Department of Psychiatry in Podgorica, it was the same, there were group conversations. I was even as chairman [unclear] on behalf of the patients. I had a notebook, we wrote there who was present, what it was, how we felt, remarks, compliments, conversations.’ (Male, 60 years old, Montenegro)

‘In a conversation when you come to the doctor for an examination that lasts 15 minutes, he usually asks you how you are feeling, what is today, so you are concentrating on what your condition is that day. It's not about, uh, talking about what your problems are and what you could possibly be working on.’ (Male, 48 years old, Serbia)

## Discussion

This study is one of few qualitative studies exploring patient participation in research projects, and the first such study conducted in SEE countries. In exploring patients’ experiences, seven themes were identified, covering a range of topics. We found a largely positive experience of LEAP participation that supported maintaining motivation and active engagement of participants, openness in communication and perception as equal partners.

Participants highlighted active and open exchange of attitudes and emotions, as well as opportunities to share their experiences and help others regarding long-term treatment. This is extremely important because mental health patients often have few social connections,^9^ feel a low sense of belonging compared with other social groups,^10,11^ and have access to less social capital compared with the general population.^12,13^ It has also been argued that actively involving patients in research projects has visible positive effects on them, including a sense of being heard and empowered, learning new skills, increased trust in research and connection with the community.^6^ Paying patients to participate in research activities can also act as a mechanism to encourage greater social inclusion.^14^ Engagement in which a person feels useful to himself and others has contributed to identification with a new social role and experience that could also help overcome the experience of inferiority or stigmatisation, and social exclusion.

The researchers found that participants often saw the meetings as therapeutic sessions. This outcome came about despite the fact that participants were seen as fellow partners throughout the LEAP process, including the data analysis process by the authors of the present study. Moreover, the LEAP leaders from all countries have explained the role of the LEAP members since the beginning of LEAP groups, which included a clear distinction between patients and advisory panellists. Participation in research as a therapeutic activity has received only limited attention in the literature, although catharsis and increased awareness of problems,^15^ as well as empowerment, treatment and sense of purpose,^16^ have been recorded. The ability to speak and express oneself freely in meetings is also an advantage, and could lead to empowerment, as evidenced by other research.^17^

Most participants did not identify challenges related to their LEAP membership. However, a small number of participants expressed weak or unclear motivation as a barrier to involvement, which may be related to their personal characteristics or the way they function. Negative cultural stereotypes about mental illnesses that produce and maintain stigma,^1^ as well as doubts about one's abilities, are usually cited as obstacles in the literature. Other participants identified the following challenges: passivity, scepticism, anxiety and physical disability. It is possible that the motivational issues of patients with negative symptoms reflected low expectations of successful performance.^18^

The participants reported the diversity of their own experiences with hospital treatment, the importance of pharmacotherapy and alliances with professionals. Attitudes were mixed, both positive and negative. They reported dissatisfaction with communication, loss of control over their lives and the negative attitude of the community toward them. On the other hand, empathic attitudes of psychiatrists were singled out as crucial for mental health patients to feel that they were ‘on the right path’ to recovery. In line with our findings, Morselli and Elgie^19^ emphasised the importance of trust, openness and respect for the relationship between patients and professionals.

The concerns and challenges associated with the COVID-19 pandemic likely affected the LEAP members’ discussion about future perspective. However, various remote mechanisms of communication (e.g. Zoom and Skype) were proposed by the participants. Interaction between the patient-led (and similar) associations in the region should be established, according to the opinions of the participants. Participants were very interested in staying involved in this or a similar panel/group. Additionally, the LEAP members could potentially contribute to the work of non-government organisations. They also expressed positive attitudes toward the implementation of digital technologies in the treatment of mental health. A systematic review that explored the feasibility of online and telephone interventions for people with psychosis supported the feasibility of such interventions, and reported a number of positive outcomes in some of the studies included, such as improved social cohesion and socialisation.^20^

### Strengths and limitations of the study

This qualitative research is the first of its kind in SSE, and the involvement of mental health patients in advisory panels is novel not only for them, but also for professionals. We hope that this study offers useful insights into involving patients in research, and might encourage further similar initiatives. The study was conducted across five countries and five languages and, as such, contributes to the field of multi-language qualitative research methodology.

However, there are limitations that need to be acknowledged. The preliminary list of codes was developed by translating five transcripts into English; the remaining transcripts were coded in the local language, and then the codes were translated into English, which may have caused some meaning to be lost in the process. However, the framework for analysing transcripts in local languages has been revised based on feedback so we feel that this limitation has been addressed.

The study was conducted during the COVID-19 pandemic, and most of the interviews were done by telephone, which limited the ability to observe nonverbal communication, and thus the influx of additional information that could complement the interpretation of the results obtained. In addition, the interviewers were also the LEAP meeting leaders, which could have led participants to provide socially desirable answers. The choice of interviews versus focus group was informed by the circumstances related to the COVID-19 pandemic, and we feel that individual interviews had a positive effect on participation rate and that this approach also facilitated participant openness and interaction.

## Data Availability

The data that support the findings of this study are available from the corresponding author, F.R., upon reasonable request.

## Supplementary material

For supplementary material accompanying this paper visit http://doi.org/10.1192/bjo.2021.1077. click here to view supplementary material
