# Supplementary material for: Experiences of patients advising on mental health research: qualitative study in South-East European countries
Source: BJPsych Open. 2022 Jan 10;8(1):e26. doi: 10.1192/bjo.2021.1077 (PMC8811779; doi:10.1192/bjo.2021.1077)
Supplement: Supplementary file 1 [file bjosup.zip › S2056472421010772sup001.docx]

**Appendix I – Topic guide for focus groups and interviews with LEAP members**

**Introduction**

The IMPULSE study is testing the (cost-) effectiveness and investigating the implementation of a psychosocial intervention for patients with psychotic disorders (DIALOG+) in low and middle income countries in South-Eastern Europe. The Lived Experience Advisory Panels (LEAP) was established to actively involve patients and carers as experts by experience to provide advice on study materials and findings. Until now the LEAP members have been part of 5 meetings regarding the IMPULSE project.

**Aim**

This topic guide has been designed to obtain and explore LEAP members’ experiences of participating in this panel and the IMPULSE research project through focus group discussions.

**Sample Composition**

All LEAP members that have been part of the panel should be included.

**Timeline**

Focus group with LEAP members will be conducted during the 5^th^ LEAP meeting during March-April 2020. The following LEAP meeting will be used to discuss findings from the focus groups in addition to other topics.

**Topic guide**

**1)**  **Introduction**

**·** Thank participants for their availability and introduce yourself and other researchers

Ø *I am XXX. I am a researcher with the IMPULSE study, and I will ask you some questions about your experiences with participating in LEAP and the IMPULSE research project.*

· Explain nature and purpose of the research

Ø *You are a member of the LEAP for the IMPULSE project. The purpose of this project is to test a new intervention called DIALOG+ which involved using a tablet with the DIALOG+ application in the routine meetings between patients and clinicians.*

Ø *DIALOG+ is an app-based therapeutic approach which aims to improve the communication between clinicians and patients and, through that, improve patients’ wellbeing. In DIALOG+, patients use a tablet to complete the DIALOG scale in the meeting with their clinicians. Based on the ratings and comparisons with previous ratings, patients decide which concerns they want to discuss in the given meeting. Each concern is then addressed with the clinician, and then the patient and clinician agree on actions/activities which will then be reviewed at the next meeting.*

Ø *Your role as a LEAP member is to provide advice on the design of the study, interpretation of results and dissemination of results as well as on other relevant project materials (e.g. Model to engage users and caregivers) as experts by experience.*

· Explain the purpose of the focus group

Ø *The purpose of this focus group is to find out more about what you experienced during your participation in LEAP and the IMPULSE research project.*

· Provide assurances about confidentiality

Ø *Your name and information will only be known by the researchers and will not be revealed to anyone else.*

· Inform participants the session will be recorded and introduce a tape recorder.

· Explain what happens to data collected – transcribing, reported, anonymity- check how they would like to be referred to during recording

Ø *The session will be audio-recorded, transcribed and analysed by the researchers and audio recordings will be destroyed once data analysis is complete. For any quotes that we use you will be identified by an ID number and all potentially identifying information will be removed.*

· Explain how the focus group works - facilitator will not say very much and will tend to ask questions, aiming to create a discussion between participants (not between participants and facilitator), all views are important, no right or wrong answers, looking for a range of views.

· Explain they should say if they are feeling stressed or uncomfortable and that they do not have to answer a question and that a topic can be changed if they need to. The focus group can be paused for a break or stopped at any time if you no longer want to continue.

· Show short video of example focus group discussion

· Discussion can last up to 1.5h

· Mobile phones are off or on silent.

· Ground rules:

Ø The most important rule is that only one person speaks at a time. There may be a temptation to jump in when someone is talking but please wait until they have finished.

Ø There are no right or wrong answers

Ø You do not have to speak in any particular order

Ø When you do have something to say, please do so. There are many of you in the group and it is important that I obtain the views of each of you

Ø You do not have to agree with the views of other people in the group

· Invite any questions before you begin.

***Notes for the group facilitator:***

**·** It is important that the subject matters in the topic guides are explored and the style of questioning kept open and explorative. Not leading.

· The focus group facilitator has a responsibility to adequately cover all prepared questions within the time allotted. S/he also has a responsibility to get all participants to talk and fully explain their answers. Some helpful general probes include:

o *“Can you tell me more about that?”*

o *“What is it like?”*

o *“What led you to (feel, think in specific way). . . “*

o *“Can you think of another example of this?”*

o *“Can you give me more details please?”*

o *“I’d like to hear more about…”*

o *“How did that make you feel?”*

o *“Can you help me understand what you mean…”*

· For group facilitation you can use the following probes:

o *“Thank you for your comment. What do others think?”*

o *“Thank you for sharing that. Can we hear some other comments?”*

**Background**

Ask participants to introduce themselves and say a little bit about themselves. Facilitators to begin and invite others.

· *We’ll start off by asking everyone to say their name and to say why they choose to be part of this LEAP.*

**2)** **EXPERIENCE OF PARTICIPATING IN LEAP AS PART OF A RESEARCH PROJECT**

For the past 1,5 years you have been involved in the research project IMPULSE as member of an advisory board called LEAP. What has been your experience with being a member of LEAP and participating in a research project? Did your experience change over time?

*Probes:*

*·*  *How much did you like or dislike conversations you had during LEAP meetings? Why?*

*·*  *What do you see as LEAP’s contribution to the project?*

*·*  *What is the benefit of participating in a group like LEAP for the service user??*

*·*  *What do you feel needs to change/improve, if anything, with the LEAP meetings? (e.g. structure of meetings, clarity about roles, responsibilities, expectations, level of support, reading materials)*

*·*  *What were the challenges, if any, with advising on research?*

*·*  *Prior to the LEAP meeting the researchers shared materials to be red in advance of the next meeting. Did you feel like you were able to complete this task before the LEAP meeting? What was easy or difficult about completing it?*

**2.** **SUSTAINABILITY AND FUTURE ASPIRATIONS**

*·*  *Would you like to continue being involved in the LEAP groups and advising on research projects?*

*·*  *What other activities would you like to see this group being involved in? (e.g. workshops, user-organisations, advocacy activities, conferences, hosting international/national visitors)*

*·*  *How would you like to see this group grow?*

**Closing remarks**

- Do you have anything else to add to what we have discussed today: any further comments or recommendations?

Thank you for your contribution.

Confirm regarding sending summaries from focus group discussion for review/future contact permissions.

*Remind participants that all information is confidential*

*Payment of 25 EUR once the focus group is completed.*

**Appendix II – Research Team and Reflexivity**

Five authors of the paper were involved in data collection and analysis: SR (male; psychologist), TM (female; psychologist), FR (female; MA student of psychology), MZ (female; psychologist), and LN (male; MSc in public health). NJ was involved in the analysis (female; psychiatrist). All researchers involved in data collection have a background in qualitative methodology and, for the purpose of this study, underwent training in qualitative interviewing and data analysis. SR is a research assistant and statistician with previous qualitative experience gained during his MA studies, the IMPULSE project, and during the preparation of his previous papers relying on qualitative methodology. TM is a coordinator of a ULO with previous experience in qualitative studies gained during her psychology studies. FR is a research assistant with previous qualitative experience gained during her MA studies. MZ is a research assistant with previous qualitative experience during her PhD studies. LN is a research assistant with experience in qualitative analysis gained through the IMPULSE study.

Regarding previous relationships with participants, researchers and LEAP members already knew each other and held 6 LEAP meetings during an 18-month period (August 2018 - December 2020). Researchers discussed their interest in exploring participants’ experiences in LEAP groups and their views about the IMPULSE project.

**Appendix III – Codes and categories**

| CATEGORIES | CODES |
| --- | --- |
| 1. Predominantly positive motivation for LEAP membership | 1.1. Motivation to be a LEAP member |
|  | 1.2. Motivation/readiness for group exchange (if ideas, opinions, attitudes)... |
|  | 1.3. Source of motivation |
|  | 1.4. Motivators |
| 2.  Therapeutic benefits from LEAP membership | 2.1. Positive emotions in general |
|  | 2.2. Positive feelings for being helpful |
|  | 2.3. Relaxation |
|  | 2.4. Social connection |
|  | 2.5. Opportunity for self-expression |
|  | 2.6. Orientation towards recovery |
|  | 2.7. Sense of group belongingness |
|  | 2.8. Mutual support |
|  | 2.9. Mutual understanding |
|  | 2.10. Free to talk |
|  | 2.11. New social role (self-perception & identification) |
|  | 2.12. Social skills development |
|  | 2.13. Boosting self-esteem |
|  | 2.14. Exchange |
|  | 2.15. Mutual trust |
|  | 2.16. Reducing self-stigma |
|  | 2.17. Wanted their voice to be heard |
| 3.  Few challenges of LEAP membership | 3.1. Skepticism |
|  | 3.2. Closeness |
|  | 3.3. Passive position |
|  | 3.4. Intrapsychic preoccupation |
|  | 3.5. Anxiety |
|  | 3.6. Social comparison |
|  | 3.7. Trust among members |
|  | 3.8. Lack of communication culture |
|  | 3.9. Retraumatisation (being in a group with patients) |
|  | 3.10. Patients' responsibility for recovery and alliance |
|  | 3.11. Issues of emotional responsiveness |
|  | 3.12. Denying challenges |
|  | 3.13. Physical disability (e.g. issues with sight) |
|  | 3.14. Discrepancy between aspirations and possibilities |
| 4.  Mostly positive evaluation of EAP meetings | 4.1. Clarity of materials |
|  | 4.2. Clarity of presentation and topic itself (during the main part of the meetings) |
|  | 4.3. Quality of leadership (approach of researchers) |
|  | 4.4. General impression on LEAP meetings |
| 5.  Various future perspectives  of  LEAP meetings | 5.1. Desired frequency and duration of meetings |
|  | 5.2. Education/training/lectures/workshops |
|  | 5.3. Cooperation with other parties |
|  | 5.4. Future challenges |
|  | 5.5. Motivation to extend their LEAP membership |
|  | 5.6. Forms of future organisation/communication |
|  | 5.7. Attitude towards inclusion of new members |
|  | 5.8. Worries about COVID-19 impact on LEAP |
| 6.  Positive attitudes towards the IMPULSE project | 6.1. Positive aspects (of the IMPULSE project / DIALOG+) |
|  | 6.2. Possible barriers/obstacles |
|  | 6.3. Familiarity with the project |
| 7.  Mixed reflexions on mental health treatment | 7.1. Patient-clinician alliance |
|  | 7.2. Previous experience with mental healthcare services and professionals |
