# Supplementary material for: Experiences of patients advising on mental health research: qualitative study in South-East European countries
Source: BJPsych Open. 2022 Jan 10;8(1):e26. doi: 10.1192/bjo.2021.1077 (PMC8811779; doi:10.1192/bjo.2021.1077)
Supplement: Supplementary file 1 [file bjosup.zip › S2056472421010772sup002.docx]

**Table 2 - Summary of themes and subthemes**

|  | **Theme** | **Subthemes** |
| --- | --- | --- |
| **1** | **Predominantly positive evaluation of LEAP meetings** | Impressions of LEAP meetings are generally positive |
|  |  | Views about the clarity of provided materials are mostly positive |
|  |  | Preferred frequency of meetings is variable |
| **2** | **Mostly high levels of motivation to participate in LEAP** | Facilitators (extrinsic and intrinsic factors) |
|  |  | Barriers (physical factors, challenges of the new role) |
| **3** | **Therapeutic benefits of participating in LEAP** | LEAP is seen as therapeutic group |
|  |  | Being in a new social role is beneficial to patients |
|  |  | Feeling free to talk |
| **4** | **Few challenges to participate in LEAP** | -- |
| **5** | **Various future perspectives of LEAP meetings** | -- |
| **6** | **Positive appraisal of the research project** | -- |
| **7** | **Mixed reflections on mental health care** | -- |
